
JOURNAL INFORMATION
==============================
NLM Title Abbreviation: Behav Neurol
Journal ID: Behav Neurol
NLM Journal ID: 8914585
Journal ID: BN

Behavioural Neurology

ISSN: 0953-4180
EISSN: 1875-8584
Publisher: Wiley

ARTICLE INFORMATION
==============================
PMCID: PMC5488617
PMID: 28698710
DOI: 10.1155/2004/259701
Article ID: 259701
Article version: 1
Subjects: Other

Editorial

Print publication date: 2004
Electronic publication date: 2004 Jun 9
Volume: 15
Issue: 1-2
First page: 1
Last page: 1
Received 2004 Jun 3; Accepted 2004 Jun 3
Copyright: Copyright © 2004 Hindawi Publishing Corporation and the authors.
Copyright year: 2004
License: This is an open access article distributed under the Creative Commons Attribution License, which permits unrestricted use, distribution, and reproduction in any medium, provided the original work is properly cited.
License URL: https://creativecommons.org/licenses/by/3.0/

==============================
### Editorial

Behavioural Neurology is restructuring its editorial management in the following way: there will be two editors of the journal, one located in the USA. and the other in the UK. The American editor will be Argye Hillis and the British editor will continue to be Andrew Mayes. Argye Hillis is a behavioral neurologist at Johns Hopkins University School of Medicine, whose research focuses on imaging and cognitive studies of aphasia and hemispatial neglect resulting from stroke or neurodegenerative disease. Andrew Mayes is a cognitive neuroscientist, whose research focuses on structural and functional imaging, lesion studies of complex forms of memory such as familiarity, recollection and priming. There will also be two new American members of the editorial board: Anjan Chatterjee, MD from the University of Pennsylvania School of Medicine in Philadelphia, and Anne Foundas from Tulane University School of Medicine in New Orleans. The aim of this restructuring is to encourage submissions from American researchers. The journal's focus will otherwise remain as it is currently stated in the Aims and Scope statement on the inside front page of each issue. We will continue to encourage Special Issues and appropriate review papers. Authors should submit their papers as email attachments and putting suggestions for possible reviewers in the email is encouraged. The reviewing and revision process will also be conducted by email. In general, authors from the Americas or Asia should submit their manuscripts to the American editor, Argye Hillis at argye@jhmi.edu and authors from Europe, Africa or Australasia should submit their manuscripts to Andrew Mayes at a.mayes@liverpool.ac.uk. If the subject matter of the paper appears to be more appropriate for either Argye Hillis or Andrew Mayes, either the author or the editor may direct it to one or the other of the editors.

Argye Hillis and Andrew Mayes
*Editors-in-Chief*
*Behavioural Neurology*

ISSN 0953-4180/04/$17.00 © 2004 – IOS Press and the authors. All rights reserved
